# Cystic adventitial disease of the popliteal artery: features on 3T cardiovascular magnetic resonance

**DOI:** 10.1186/1532-429X-10-38

**Published:** 2008-08-13

**Authors:** Anderanik Tomasian, Chi Lai, J Paul Finn, Hugh Gelabert, Mayil S Krishnam

**Affiliations:** 1 Department of Radiological Sciences, David Geffen School of Medicine, University of California, Los Angeles, CA, USA; 2 Department of Pathology, David Geffen School of Medicine, University of California, Los Angeles, CA, USA; 3 Department of Surgery, David Geffen School of Medicine, University of California, Los Angeles, CA, USA; 4 Suite 3371, Peter V. Ueberroth Bldg, 10945 Le Conte Ave, Los Angeles, CA, 90095-7206, USA

## Abstract

Cystic adventitial disease (CAD) of the popliteal artery is a rare vascular disease of unknown etiology in which a mucin-containing cyst develops in the adventitial layer of the artery. We report the case of a 26-year-old male with CAD of the right popliteal artery diagnosed non-invasively with 3 Tesla cardiovascular magnetic resonance and confirmed on post-operative histopathology.

## Introduction

Cystic adventitial disease (CyAD) is a rare non-atherosclerotic condition which results in intermittent claudication due to peripheral vascular insufficiency caused by compression of the arterial lumen by a cystic collection of mucinous material containing varying combination of mucopolysaccharides and mucoproteins within the adventitial layer of the artery. The disease predominantly affects the popliteal artery (85% of cases), typically in young to middle-aged men with a male-to-female ratio of 15:1 [1]. The etiology of CyAD remains controversial [1]. Recent advances in cross-sectional imaging have made possible non-invasive diagnosis of this disease using computer tomography (CT) or cardiovascular magnetic resonance (CMR) [2,3].

## Case report

**Figure 1:**
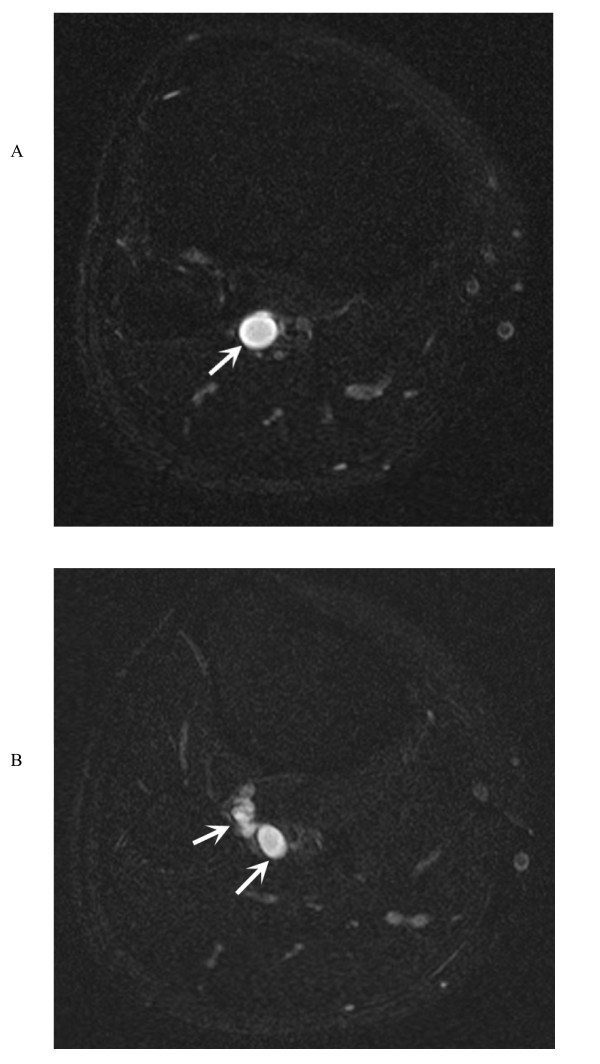
Axial T2-weighted fat saturated turbo spin echo images demonstrate a well defined, homogeneously hyper-intense multi-lobulated cystic lesion in the right popliteal fossa (Figure 1a and 1b, arrows).

**Figure 2:**
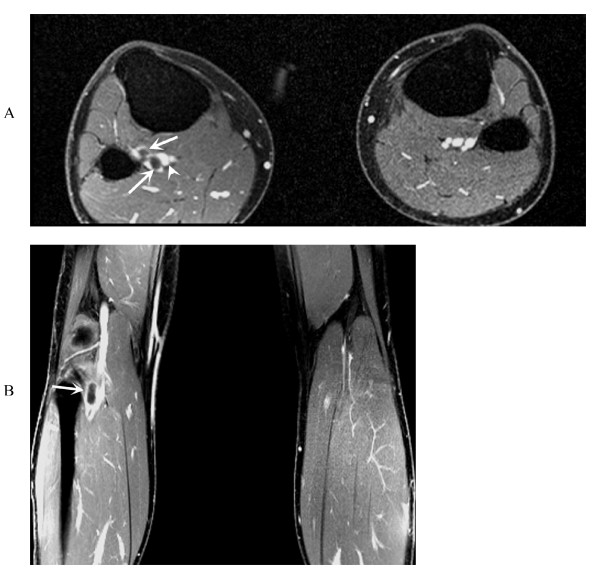
**Axial and coronal T1-weighted post-contrast fat saturated gradient echo (GRE) images show no evidence of contrast enhancement of the well defined hypo-intense cystic mass.** Compression of the popliteal arterial lumen (figure 2a and 2b, arrows), and normal enhancement of the popliteal vein (figure 2a, arrowhead) are noted.

**Figure 3:**
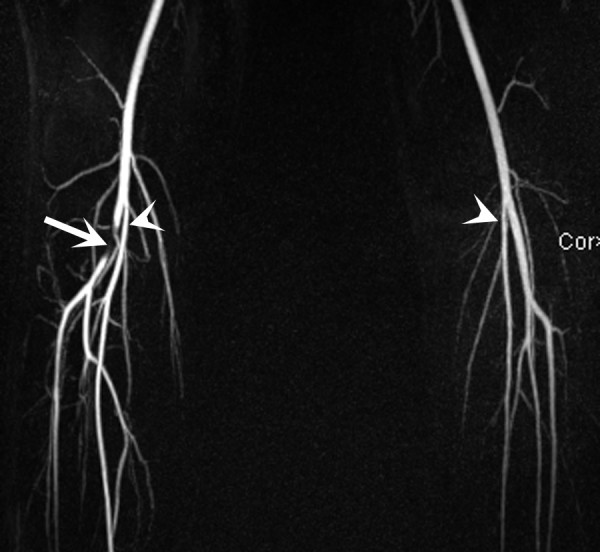
**Maximal intensity projection (MIP) reconstruction image from high spatial resolution contrast enhanced MR angiography reveals a long segment focal near occlusion of the right popliteal artery (arrow)**. Note is made of high take-off of the bilateral posterior tibial arteries (arrowheads).

A 26-year old male presented with a 15-month history of progressive right calf intermittent claudication which was more severe on stairs. The maximum walking distance was approximately 50 meters at the time of presentation. The patient had no risk factors for vascular disease such as hypertension, diabetes mellitus, hyperlipidemia, and smoking. Physical examination revealed mild tenderness at the superio-medial aspect of the right calf, normal femoral, popliteal, posterior tibial, and diminished dorsalis pedis pulses, and no neurologic deficit in the right lower extremity. Based on history and physical examination, patient was referred for CMR and high resolution contrast enhanced MR angiography (CE-MRA) of the lower extremity to assess popliteal artery entrapment syndrome. CMR was performed on a 3 Tesla MR system (Magnetom Tim Trio, Siemens Medical Solutions, Erlangen, Germany) (Table 1). Pre-contrast T1-weighted Spin-echo and T2-weighted Turbo Spin-echo images demonstrated a multilobulated cystic lesion within the wall of the popliteal artery smoothly compressing the adjacent arterial lumen with homogeneous low- and high-signal intensities on T1- and T2-weighted images, respectively (Figure 1). In addition, T1-weighted post-contrast GRE images showed no evidence of peripheral or central enhancement of the well-defined hypo-intense cystic mass (Figure 2). CE-MRA (at rest and with plantar/dorsi-flexion) showed extrinsic compression of the right popliteal artery with resultant near occlusion over a distance of 4 cm below the knee joint (Figure 3). Distal to this lesion the popliteal artery was reconstituted by collaterals. The findings favored the diagnosis of CyAD of the popliteal artery. There was no evidence of popliteal entrapment syndrome or deep vein thrombosis.

**Table 1:** Imaging Sequences and Parameters for Cardiovascular Magnetic Resonance

**Pulse sequence**	**TE (msec)**	**TR (msec)**	**Slice thickness (mm)**
T1-weighted Spin-echo (axial plane)	14	750	5
T2-weighted Turbo Spin-echo (axial plane)	86	4000	4
T1-weighted post-contrast fat saturated two-dimensional gradient echo (GRE) (axial and coronal planes)	2.1	243	3
CE-MRA: three dimensional fast GRE*	1.1	2.9	0.8

*Generalized Autocalibrating Partially Parallel Acquisition or GRAPPA factor of 4 was applied. CE-MRA was performed following intravenous administration of 15 mL of contrast (Magnevist, Berlex Laboratories, Wayne, New Jersey, USA) at the rate of 1.2 mL/second followed by 30 mL of saline at the same rate.

**Figure 4:**
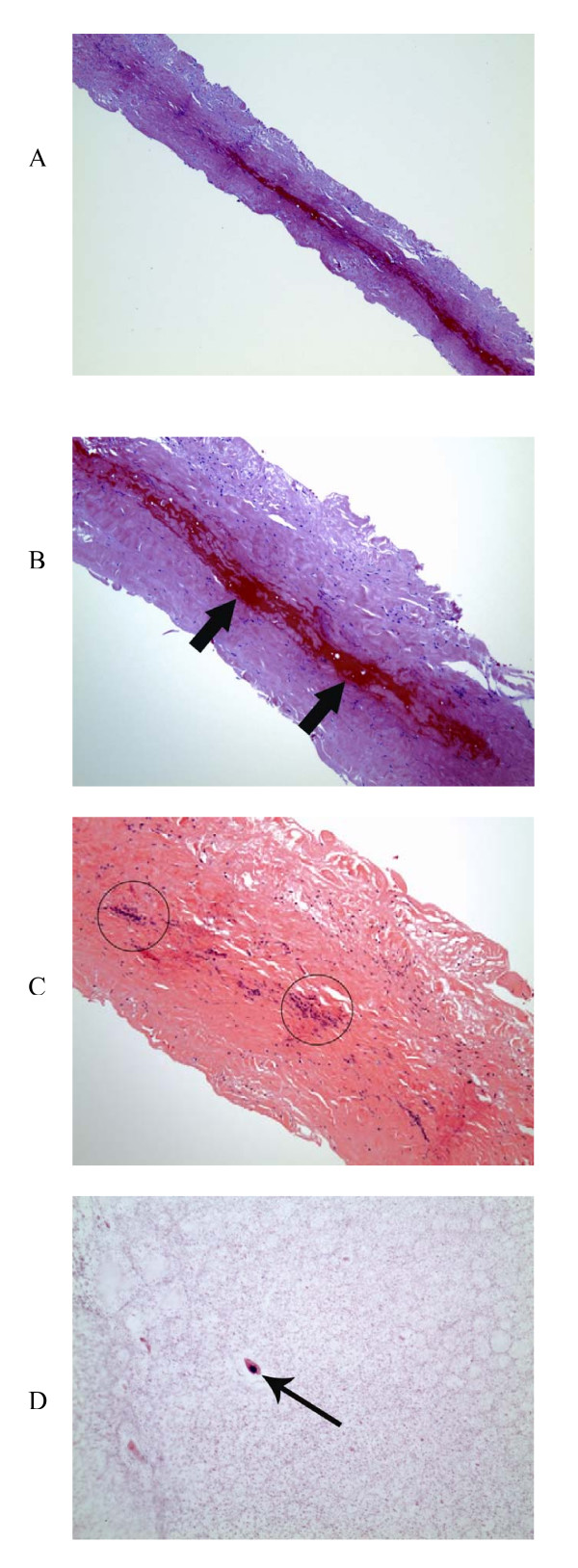
**(a) The cyst wall is comprised of dense, fibrous tissue, which is consistent with the adventitia of a blood vessel (H&E stain; 40×, original magnification)**. **(b) **At higher magnification (H&E stain; 100×, original magnification), foci of hemorrhage is seen within the cyst wall (arrows). **(c) **There were also focal areas of chronic inflammation within the cyst wall (circled areas) [H&E stain; 100×, original magnification]. **(d) **The cyst contents consist mostly of proteinaceous debris with rare, scattered degenerated macrophages (arrow) [H&E stain; 400×, original magnification].

The patient underwent open resectional cystotomy two weeks later. The popliteal artery was exposed from a posterior approach through a longitudinal incision. A 4-cm length of the artery was observed to be grossly enlarged. The cyst was punctured and after evacuating the clear gelatinous fluid, the wall was resected with Potts scissors. With release of the cyst compression, arterial pulse distal to the lesion improved immediately. Histopathology confirmed cystic adventitial disease as evidenced by dense fibrous adventitial tissue of the intramural cyst wall and the mucoproteinaceous contents (Figure 4).

The patient returned to full activity in one week, and remained asymptomatic during the 20-month follow-up period.

## Discussion

The most common non-atherosclerotic diseases of the popliteal artery include thrombosed aneurysm, embolism, entrapment syndrome, and CyAD [4]. The incidence of CyAD is estimated to be 1 in 1200 cases of claudication [1], with cases described involving external iliac, femoral, popliteal, radial, and ulnar arteries and veins [2-5]. The symptoms usually include progressive claudication of the lower extremities with no significant evidence of atherosclerotic disease, and although CyAD usually presents in middle-aged patients [1], a few reports exist featuring this condition in other ages [6,7]. The etiology of CyAD is unclear, and the proposed hypotheses include repetitive trauma to the adventitia caused by flexion injuries leading to cystic degeneration, embryological origin, direct communication with the herniated synovial structures of the adjacent joint, and CyAD as a part of a connective tissue disease [8]. Slow progression of CyAD over a period of several years accounts for masslike appearance and large size of the most lesions [4].

As an inexpensive and readily available diagnostic modality, ultrasound demonstrates anechoic or hypoechoic cystic lesions on gray-scale images, and intra-arterial sonogram reveals adventitial origin of the lesion [3]. Although regarded as the gold standard, conventional angiography has disadvantages such as invasiveness, and exposure to radiation and nephrotoxic contrast agents, and is only diagnostic when characteristic findings such as scimitar (eccentric compression) or hourglass (concentric compression) signs are present [3]. CT and CMR are excellent non-invasive diagnostic modalities for accurate characterization of the cystic lesions and their anatomical relationship to vascular structures [2,3,9]. Contrast enhanced CT may demonstrate CyAD as a non-enhancing cystic mass extrinsically compressing the enhancing crescentic arterial lumen [10].

The introduction of 3T MR systems, with higher inherent signal-to-noise ratio compared to lower field strength scanners, has resulted in improved spatial resolution and faster data acquisition, and with the heightened signal sensitivity to gadolinium-based contrast agents at 3T, high resolution CE-MRA can be performed with low dose contrast protocols, further improving non-invasive assessment of vascular diseases.

The CMR features of CyAD are quite characteristic, and the anatomical extent of the arterial intramural cystic lesions are demonstrated using multi-planar data acquisitions. Individual cysts typically show homogeneous low signal intensity on T1-weighted Spin-echo, and high signal intensity on T2-weighted Turbo Spin-echo images [9], as described in our case. On post-contrast T1-weighted GRE images the non-enhancing hypo-intense intramural cysts with smooth extrinsic compression of the adjacent arterial lumen are clearly depicted. However, since the underlying pathophysiology leads to myxoid degeneration [1], lesions may demonstrate enhancement as seen in other myxoid processes. High resolution CE-MRA at 3T is a robust technique for accurate assessment of degree and length of the arterial stenosis or occlusion while depicting lack of significant atherosclerotic process in other arteries of the extremities in CyAD, and may alleviate the need for invasive catheter angiography. Since CyAD may be bilateral, it is important to image both legs. Follow up imaging with CE-MRA or high resolution ultrasound may be performed to look for recurrence or any residual disease.

Surgical treatment options to restore popliteal arterial flow include resection of the affected artery and interposition grafting [11], aspiration of cystic contents [12], and resectional adventitial cystotomy [3].

## Conclusion

Utilizing various high resolution image acquisition sequences in multiple orientations combined with CE-MRA at 3T, CMR has an important role for non-invasive morphological assessment, tissue characterization, and vascular evaluation in cystic adventitial disease.

## Consent

Written informed consent was obtained from the patient for publication of this case report and any accompanying images. A copy of the written consent is available for review by the Editor-in-Chief of this journal.

## Competing interests

The authors declare that they have no competing interests.

## Authors' contributions

AT, C.L, JPF, HG, MSK Data acquisition, or analysis and interpretation, AT, CL, JPF, HG, MSK Critical revision for important intellectual content, AT, MSK Manuscript drafting, AT, CL, JPF, HG, MSK Final approval of the manuscript, HG Surgical procedure.
